# Insights into the interplay between gut microbiota and lipid metabolism in the obesity management of canines and felines

**DOI:** 10.1186/s40104-024-01073-w

**Published:** 2024-08-08

**Authors:** Kaiqi Li, Xiangyu Xiao, Yuling Li, Sichen Lu, Jianghang Zi, Xiaoqiang Sun, Jia Xu, Hao-Yu Liu, Xiaoqiong Li, Tongxing Song, Demin Cai

**Affiliations:** 1 Laboratory of Animal Physiology and Molecular Nutrition, Jiangsu Key Laboratory of Animal Genetic Breeding and Molecular Design, College of Animal Science and Technology, Yangzhou University, Yangzhou, 225009 China; 2 College of Animal Science and Technology, Huazhong Agricultural University, Wuhan, 430070 China; 3 School of Life Science and Engineering, Foshan University, Foshan, 528231 China; 4 State Key Laboratory for Managing Biotic and Chemical Threats to the Quality and Safety of Agro-Products, Institute of Food Science, Zhejiang Academy of Agricultural Sciences, Hangzhou, Zhejiang 310021 People’s Republic of China; 5 College of Agriculture, Jinhua Polytechnic, Jinhua, 321017 China

**Keywords:** Cat, Dog, Gut microbiota, Lipid metabolism, Obesity Management

## Abstract

Obesity is a prevalent chronic disease that has significant negative impacts on humans and our companion animals, including dogs and cats. Obesity occurs with multiple comorbidities, such as diabetes, hypertension, heart disease and osteoarthritis in dogs and cats. A direct link between lipid metabolism dysregulation and obesity-associated diseases has been implicated. However, the understanding of such pathophysiology in companion animals is limited. This review aims to address the role of lipid metabolism in various metabolic disorders associated with obesity, emphasizing the involvement of the gut microbiota. Furthermore, we also discuss the management of obesity, including approaches like nutritional interventions, thus providing novel insights into obesity prevention and treatment for canines and felines.

## Introduction

Having cats and dogs as companions is believed to contribute to good physical health and psychosocial well-being of humans; hence, the importance of pets in our lives is becoming obvious [1]. Meanwhile, the prevalence of obesity and associated metabolic diseases is increasing in companion animals. This is closely related to the dysregulation of lipid metabolism, including the biosynthesis and degradation of lipids such as fatty acids (FAs), triglycerides, cholesterol, etc. [2]. While emerging evidence suggests that gut microbiota plays a major role in regulating the host’s lipid metabolism, by producing short-chain fatty acids (SCFAs), which serve as both substrates and modulators. On the one hand, SCFAs especially acetate and butyrate can be converted into acetyl-CoA, thereafter enter the tricarboxylic acid cycle and/or synthesize palmitic acid and other lipids. On the other hand, it is shown in mice and humans that butyrate could promote FA oxidation and stimulate the metabolic activity of brown adipose tissue, subsequently preventing diet-induced obesity and hypertriglyceridemia [3]; gut microbiota can also reshape the host-derived bile acids (BAs) pool by chemically modify them into secondary BAs; as well as regulating host satiety, affecting gene expression, maintaining intestinal barrier function, modulating immune responses, and producing specific enzymes etc. These various processes collectively impact the host's energy expenditure, lipid absorption and metabolic pathways, potentially contributing to the development of metabolic diseases such as obesity [4, 5]. The causal role of gut microbiota in host lipid metabolism dysregulation is best demonstrated in germ-free mice where they develop obesity when colonized with “obese microbiota” [6]. Moreover, recent studies have revealed that the gut microbiome may regulate intestinal epithelial lipid absorption and white adipose tissue lipid accumulation through an array of non-coding RNAs [7, 8]. However, how these lipid metabolism and gut microbiota interact in companion animals is still poorly understood.

In this review, we provide an overview of the occurrence, development, and outcomes of obesity in dogs and cats. Specifically, we aim to elucidate the mechanisms of lipid dysregulation, its connection with obesity-related complications in dogs and cats, and the differences between the two species. Furthermore, we illustrate the potential interaction of lipid metabolism and gut microbiota. In addition, we also discuss obesity management in dogs and cats from a nutritional perspective, opening up new avenues for obesity prevention and intervention in dogs and cats.

## Epidemiological and etiological studies of obesity in dogs and cats

### Prevalence of obesity in dogs and cats

Similar to humans, obesity is also widespread in domestic cats and dogs. Early studies conducted in the U.S. and other countries have demonstrated that the prevalence of obesity among dogs is between 25% and 40.5%, whereas among cats, it ranges from 6% to 52% [9–13]. This incidence has increased sharply in recent decades [14–17]. Moreover, the prevalence of comorbidities related to obesity has risen steadily over the past few years. This trend has even surpassed that observed in humans, posing a serious concern within the veterinary community [5, 13, 18]. Therefore, understanding the diagnostic criteria and causative factors of obesity in dogs and cats is crucial for effective management.

### Obesity assessment criteria for dogs and cats

In the clinical context, dogs and cats are classified as being overweight or obese when their body weight surpasses the ideal body weight for their body size by 15% or 30%, respectively [19]. However, due to body type, age, and breed variations, this method is highly error-prone when assessing whether pets are obese. Currently, the most accurate method for determining obesity in dogs and cats is measuring their fat and muscle mass using dual-energy X-ray absorptiometry (DEXA) [20]. However, DEXA requires sedation or anesthesia, making it uneconomical and impractical for daily assessment of obesity in dogs and cats. The most widely used method for diagnosing obesity in dogs and cats is the 9-point body condition score (BCS), developed and validated by researchers at Purdue University in 1997 [21]. Compared to body mass index (BMI), which is used for assessing human weight, BCS is used to quantify excess body mass and can give an estimation of excess body fat without measuring weight and height. The ideal BCS is 5, while a BCS of 6 or 7 correlates to overweight and obesity may be identified based on a BCS of 8 out of 9 [22]. Although the evaluation criteria are subjective and depend on factors such as visual and tactile perception, the accuracy of the scoring system is not compromised. Some studies have shown that BCS strongly correlates with DEXA measurement of body fat content [23, 24]. Besides, this scoring system has been incorporated into determining the nutritional requirements of dogs and cats by the National Research Council of the National Academies for calculating their energy needs [25]. Therefore, BCS offers an accurate and convenient approach to assess obesity in cats and dogs.

### Major contributing factors to obesity in dogs and cats

To effectively treat obesity in dogs and cats, it is necessary to establish criteria to determine the condition and understand the underlying factors contributing to its onset. Various factors can be classified broadly into two categories: extrinsic/environmental factors and intrinsic factors. For dogs, the primary risk factors for obesity include breed predisposition, age, gender, neutering, and ad libitum feeding [26–28]. For cats, in addition to these factors, physical inactivity is also one of the causes of feline obesity [15, 16, 29, 30]. Nowadays, not only diet but also its quality is considered relevant in the development of obesity, i.e., the overall nutritional composition and balance of a diet, including the presence and proportions of essential nutrients. Although it is hard to measure, the concept of diet quality has garnered substantial attention in nutritional research and is closely related to the quality of life. Specifically, a poor-quality diet characterized by excessive caloric intake, imbalanced nutrient composition, and low nutrient density can cause weight gain and obesity in pets [31–34]. Finally, neutering is of importance for pet obesity since the procedure lowers the metabolic rate of dogs and cats; an increase in food intake will lead to greater susceptibility to obesity in these animals, particularly those who lack exercise [28, 35, 36]. Although pet owners have the means to effectively address these two factors (exercise and ad libitum feeding), some owners may consider a plump appearance endearing, failing to recognize the seriousness of pet obesity as a significant concern [37, 38]. Thus, it is necessary for us to clarify the issue so pet owners can comprehend the detrimental outcomes that obesity can have on their pets.

## Outcomes of obesity in dogs and cats

**Fig. 1:**
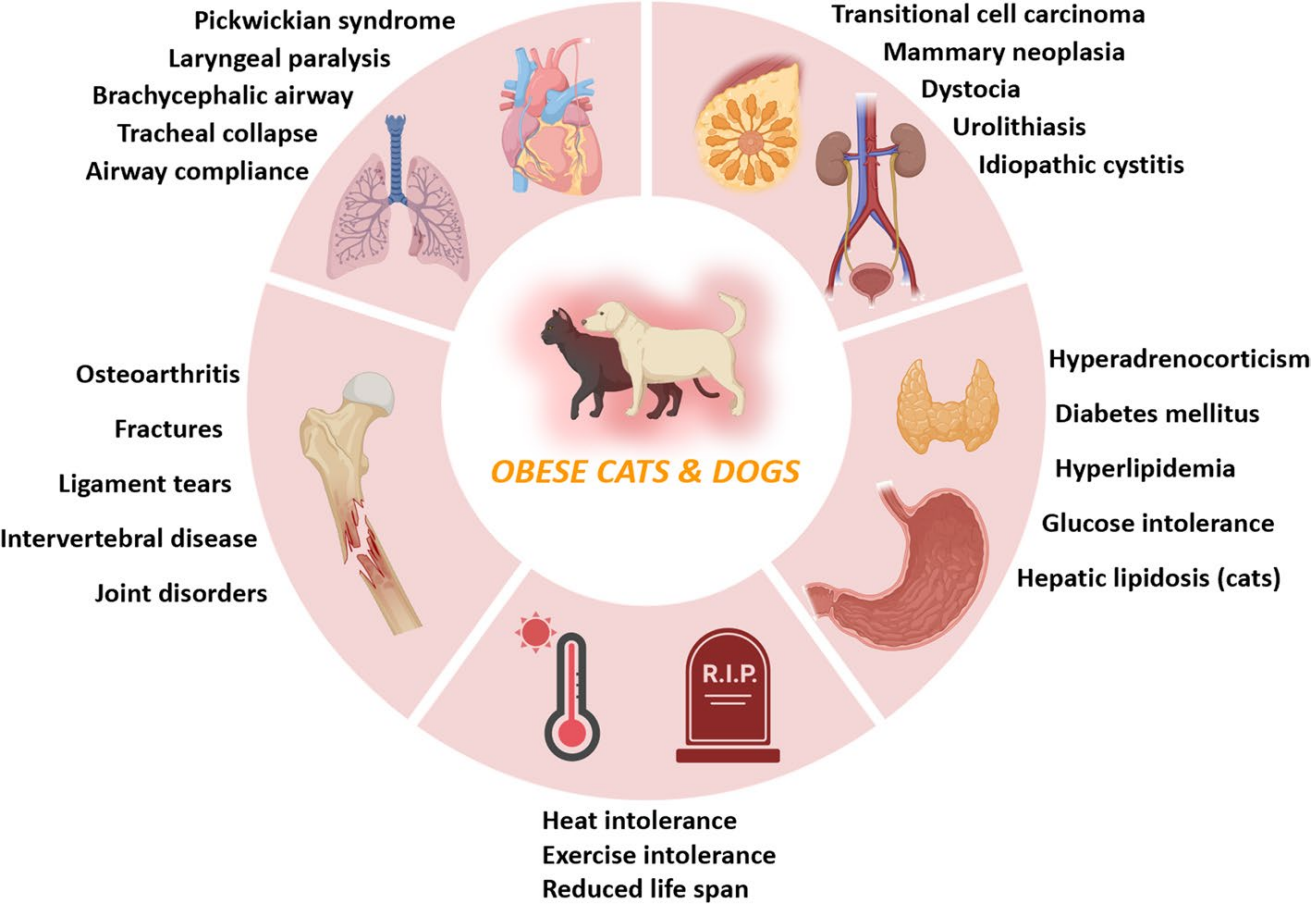
Obesity complications in obese dogs and cats. Overweight dogs are prone to a higher incidence of heart and lung diseases, hypertension, reproductive disorders, and surgical complications. Similarly, obesity in cats is associated with an increased incidence of endocrine and metabolic disorders such as diabetes, hepatic lipidosis, and lameness

Obesity is a major chronic disease in humans with multiple comorbidities, such as diabetes, hypertension, and coronary heart disease [39]. Similarly, it significantly impacts the health of dogs and cats, increasing their mortality (Fig. 1).

Overweight and obese dogs are also prone to have a higher incidence of heart and lung diseases, hypertension, reproductive disorders, and surgical complications [27]. Obesity in cats is associated with an increased incidence of endocrine and metabolic disorders such as diabetes, hepatic lipidosis, and lameness [40].

### Diabetes mellitus

Diabetes mellitus (DM) in canine and feline is one of the metabolic diseases caused by genetic predisposition and comes along with obesity, where the animals exhibit insufficient insulin secretion and resistance [41]. Animals with DM could suffer from polydipsia, polyphagia, and polyuria, and start to show weight loss, physical decline, and proteinuria. The accumulation of excess fat and lipid metabolism disorders from obesity leads to insufficient insulin secretion or decreased insulin sensitivity, resulting in increased blood glucose levels in animals and ultimately leading to the occurrence and development of DM [41]. DM in dogs and cats shares several resemblances with human DM. However, it is essential to highlight that there are differences between these species. The effects of insulin resistance in dogs and cats are different. In obese dogs, the impact of a large amount of fat is more insulin resistance, resulting in increased insulin secretion in the first stage. In obese cats, the first stage of insulin secretion will be missing or reduced due to poor glucose tolerance caused by a large amount of fat [42, 43]. However, the final result is type 2 diabetes. Moreover, while obese dogs may display insulin resistance, this does not necessarily lead to the onset of DM [44]. It is worth noting that diabetes in dogs typically presents as a type 1 diabetes-like syndrome. The etiology underlying β-cell destruction in diabetic canines is often elusive, albeit evidence suggests that immunity-mediated destruction of β-cells akin to type 1 diabetes in humans frequently contributes to the explication of pathogenesis [45, 46]. In contrast, overweight cats often develop diabetes mellitus, which shares similarities with human type 2 diabetes [47]. Concurrent with the insulin resistance in DM, dysfunctions in amylin secretion and particularly in the processing of amylin that results in its deposition as amyloid in the islets could potentially account for the gradual demise of β-cells [48]. As a naturally occurring amyloidogenic protein in several animal species, feline islet amyloid polypeptide plays a critical role in β-cell apoptosis [26]. Unlike obese humans and cats, insulin resistance in obese dogs does not spontaneously progress to type 2 DM, because the amylin protein in dogs does not aggregate and form pancreatic islet amyloid [49, 50]. Together, obesity-induced DM is one of the critical metabolic diseases in dogs and cats.

### Hepatic lipidosis

Obesity not only leads to diabetes but also increases the risk of hepatic lipidosis. Hepatic lipidosis is identified by the atypical buildup of triglycerides in over 80% of hepatocytes [51, 52]. This accumulation leads to a secondary impairment of liver function and intrahepatic cholestasis [53, 54]. Low-density lipoprotein (LDL) content is significantly higher in cats with hepatic lipidosis than in healthy cats [55, 56].

Hepatic lipidosis is the most common hepatobiliary disease and has been more widely studied in cats than dogs [54, 57, 58]. Meanwhile, in dogs, researchers generally use the term “diffuse vacuolar hepatopathy” to refer to this type of disease rather than "hepatic lipidosis" [59]. Hence, the present section only focuses on feline hepatic lipidosis. Some studies have observed that the progression of hepatic lipidosis is most prompt and consistent when felines exhibit elevated BCS [57]. Feline hepatic lipidosis is more prevalent and often results from an underlying disease, including DM, pancreatitis, or renal failure [60]. The pathophysiology of feline hepatic lipidosis is complex and not yet fully illustrated. Targeting lipid metabolism will help to ameliorate obesity and its related hepatic lipidosis.

### Other complications of obesity in cats and dogs

In dogs and cats, aside from the development of diabetes and hepatic lipidosis, obesity is also associated with the incidence of other diseases, impacting cardiac function negatively [61]. Compared with lean canines, corpulent canines exhibit an elevation in heart rate and a mild to moderate augmentation in blood pressure [62, 63]. It is well-established that obese cats tend to display a higher heart rate and greater cardiac muscle contraction compared to their average-sized counterparts [64]. Additionally, obesity leads to the accumulation of large amounts of adipose tissue, infiltrating the atrial tissue [65].

Obese felines and canines have been demonstrated to have an increased vulnerability to urolithiasis, which is correlated with elevated food consumption, adipose tissue accumulation, and a heightened excretion of minerals within the urinary system [66]. Urolithiasis, commonly called urinary stone disease, is characterized by calculi formation within the urinary system [67]. Based on their location within the urinary tract, the calculi are categorized as nephroliths (in the kidneys), ureteroliths (in the ureters), urocystoliths (in the bladder), and urethroliths (in the urethra) [66]. Osteoarthritis is a debilitating condition marked by the gradual deterioration of articular cartilage, resulting in the loss of both proteoglycans and collagen [68]. Additionally, subchondral bone sclerosis, periarticular osteophyte formation, and chronic synovial membrane inflammation further exacerbate the disease's progression [68]. Although various factors such as age, gender, and breed of animals can contribute to the development of osteoarthritis, obesity stands out as a particularly influential risk factor [69]. Obesity increases mechanical stress on the cartilage and leads to a higher incidence of non-weight-bearing joint involvement in osteoarthritis [70]. Obesity poses various risks to dogs and cats, suggesting that its treatment is a tremendous priority in pet health.

## Association between obesity and lipid metabolism dysregulation in pets

### Comparison of lipid metabolism in dogs and cats versus other animals

#### De novo lipogenesis

**Fig. 2:**
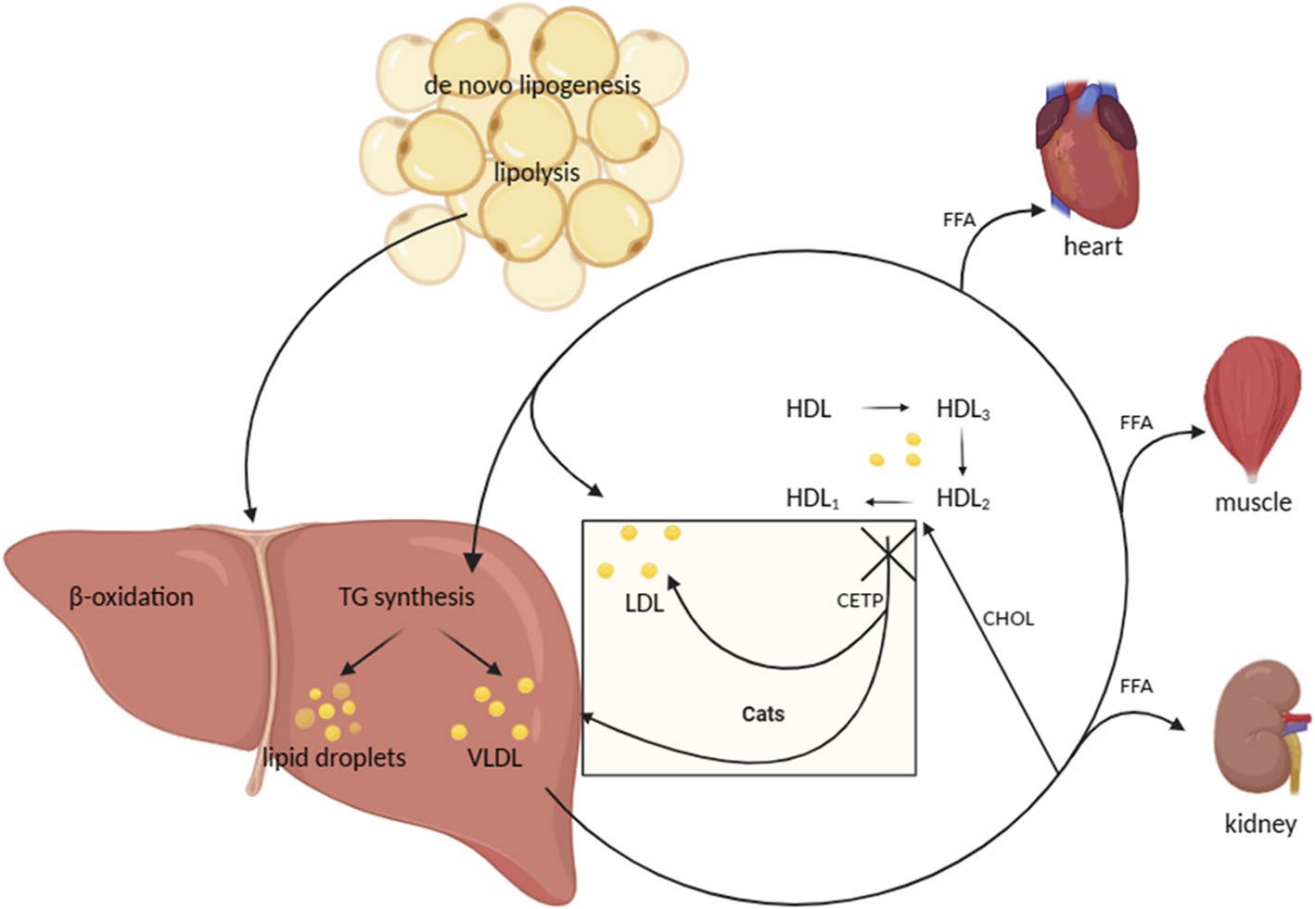
Differences in lipid metabolism underlying diabetes mellitus in obese dogs versus cats. In canine, the lack of CETP enzyme causes a perpetual acquisition of cholesteryl esters by HDL2 molecules, ultimately leading to the generation of distinctive HDL1 molecules. Next, HDL1 facilitates the transfer of cholesteryl esters from tissues to the liver for either disposal or reuse rather than to LDL or VLDL molecules that, in humans, transport cholesterol to peripheral tissues. TG: triglyceride, VLDL: very low-density lipoproteins, LDL: low-density lipoproteins, HDL: high-density lipoproteins, CETP: cholesterol ester transfer protein, FFA: free fatty acid

De novo lipogenesis (DNL) or de novo FA synthesis is a metabolic pathway that generates fatty acids from surplus carbohydrates. These fatty acids can be incorporated into triglycerides (TGs) for energy storage [71]. Under normal conditions, DNL occurs primarily in the liver and adipose tissue and is believed to play a major role in maintaining serum TG homeostasis [72]. In canines and felines, DNL predominantly occurs in adipose tissues, whereas in humans and rodents, the liver is the major site [73] (Fig. 2). Acetate is considered to be the primary substrate for DNL in feline [74]. Furthermore, it has previously been shown in organoids derived from the livers of mice, humans, dogs, and cats when administered with FA, lipid accumulation can be detected in all species. Notably, feline liver organoids exhibited a greater propensity for lipid droplet accumulation than their human counterparts [75]. It is possible that the liver of cats has a relatively weak capacity for lipid metabolism. Some researchers speculate that the other metabolic pathways handling excess FFA (β-oxidation, very low-density lipoprotein secretion) are quickly saturated in feline hepatocytes, leading to extensive lipid-droplet formation [75]. Owing to the inefficiency of the feline liver in metabolizing FA originating from abundant peripheral lipolysis, a buildup of lipids transpires in the liver [55]. In addition, dogs, being descendants of wolves, have evolved to adeptly manage the fluctuating nutrient availability associated with feast and famine eating patterns. This adaptation has endowed them with superior resilience in coping with nutritional extremes [76]. Therefore, compared to cats, dogs are generally less susceptible to developing "hepatic lipidosis".

#### Lipoprotein metabolism

Obesity is correlated with dysregulation of lipid metabolism. Studies have demonstrated that overweight neutered cats exhibit elevated serum non-esterified fatty acids (NEFAs), triglycerides, and cholesterol compared to their lean counterparts during fasting [77]. These aqueous insoluble lipids are transported through plasma in particular particles called lipoproteins. Chylomicrons, which are large lipoproteins rich in triglycerides, are assembled around apoB48 in enterocytes of the small intestine and transport dietary lipids [78]. In contrast, very low-density lipoproteins (VLDL), which are smaller in size, contain apoB100 and are produced by hepatocytes [79]. High-density lipoproteins (HDLs) are primarily synthesized in the liver and play a crucial role as donors and recipients of apolipoproteins C, apo E, and various lipids from other circulating lipoproteins [80]. HDLs also have a critical function in the reverse cholesterol transport pathway by transferring cholesterol from peripheral tissues to small circulating discoid HDL molecules, which leads to the conversion of these molecules into nascent HDL_3_ molecules [81, 82]. In canine, the lack of cholesterol ester transfer protein (CETP) enzyme, which is present in humans, causes a perpetual acquisition of cholesteryl esters by HDL_2_ molecules, ultimately leading to the generation of distinctive HDL_1_ molecules [83] (Fig. 2). Next, HDL_1_ facilitates the transfer of cholesteryl esters from tissues to the liver for either disposal or reuse rather than to LDL or VLDL molecules that, in humans, transport cholesterol to peripheral tissues [59]. It has been proposed that this role of HDL_1_ contributes to the lower prevalence of atherosclerotic disorders in dogs relative to humans [84]. Although CETP enzyme deficiency is also present in cats, it does not lead to the formation of HDL_1_ particles as observed in dogs [80, 85]. It is also observed that atherogenesis and coronary artery disease are not a feature of feline obesity or diabetes [80, 86]. The precise mechanism underlying this phenomenon has not yet been comprehensively investigated.

#### Adipokines

Obesity in cats and dogs has been associated with alterations in adipokines [87, 88]. Currently, the most studied adipokine in cats and dogs is adiponectin. Fully differentiated adipocytes exclusively synthesize adiponectin and circulate in the plasma as multimers, with the high molecular weight isoform exhibiting the most significant biological activity [26]. In felines, the expression of the adiponectin gene is markedly elevated in visceral adipose depots compared to other depots [89, 90]. Some findings have indicated that high molecular weight (HMW) multimers constitute approximately 80% of total adiponectin in cats, whereas humans exhibit only 30% HMW adiponectin [91, 92]. Similarly, dogs possess a higher concentration of HMW adiponectin than humans [93]. HMW multimers, strongly related to insulin sensitivity and body fat mass, show more excellent activity compared to total adiponectin [92]. In addition, adiponectin has profound effects on increasing insulin sensitivity and decreasing lipid concentrations, as well as anti-inflammatory and anti-atherosclerotic properties [94–96]. This could be a potential explanation for the lower susceptibility of dogs and cats to atherosclerosis. In humans, reduced levels of circulating total or HMW adiponectin are associated with insulin resistance and are indicative of the onset of type 2 diabetes or other metabolic syndrome [97]. Some studies have failed to find a correlation between obesity and adiponectin concentrations in dogs despite significant evidence of obesity’s effects on insulin resistance [88, 98, 99]. Unlike in humans, obese dogs do not experience a decrease in HMW adiponectin levels, nor do the levels change after losing weight [93, 99]. It has been suggested that this species’ difference in adiponectin may be a protective factor against developing type 2 diabetes in dogs since this condition does not occur naturally in this species [93].

### The gut microbiota associated with obesity and its regulatory mechanism

**Fig. 3:**
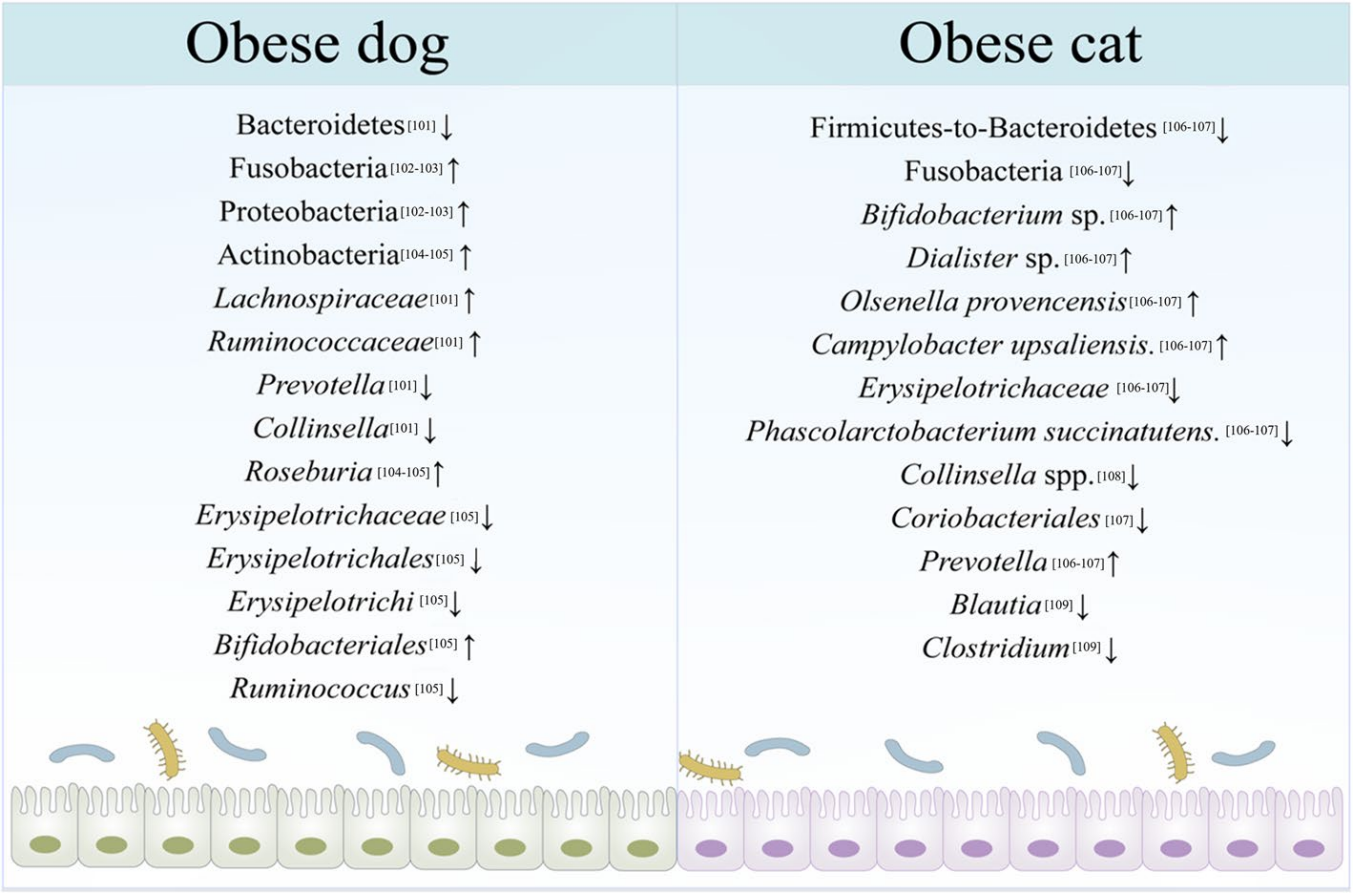
Altered gut microbiota in obese dogs and cats

Recent studies have consistently established a strong association between gut microbiota dysbiosis and the onset and progression of obesity [100]. Evidence of significant differences in the composition and biodiversity of gut microbiota between obese and healthy pet animals is also accumulating (Fig. 3). In dogs and cats, five major phyla have been identified: Firmicutes, Fusobacteria, Bacteroidetes, Proteobacteria, and Actinobacteria [5, 13].

#### Gut microbiota alteration in obese dogs

A study of 20 dogs reveals that the relative abundance of Firmicutes was lower in obese dogs when compared to the lean ones, including a decrease of *Clostridiaceae* (affiliated with Firmicutes). Meanwhile, the number of Bacteroidetes was higher in obese dogs, mainly due to the increase of *Bacteroidaceae.* Interestingly, the relative abundance of both Fusobacteria and Proteobacteria was increased in the gut of obese dogs, while these bacteria were reduced when obese animals lost weight. Additionally, it is also found that the abundance of *Coriobacteriaceae*, which belongs to the Actinobacteria phylum, was reduced in obese dogs, whereas in obese dogs lost weight, its abundance was then increased to the level in the gut of lean dogs [101]. Another study also shows a significant increase of *Proteobacteria* and *Fusobacteria* [102] in the gut microbiota of obese dogs compared to the lean ones. While the latter gut microbiota is predominantly dominated by Firmicutes [103]. Consistently, a study of 21 obese and 22 lean dogs shows a higher relative abundance of Actinobacteria and *Roseburia* in lean dogs’ gut microbiota compared to obese dogs [104]. In contrast, an analysis of 66 clinically healthy dogs’ fecal samples using 16S rRNA gene sequencing reveals a higher proportion of *Bifidobacteriaceae* (affiliated with Actinobacteria) in obese dogs. At the genus level, *Blautia*, *Lachnospiraceae*, and *Eubacterium biforme* are increased in the normal weight dogs, while the abundance of *Ruminococcus* was decreased in obese dogs [105]. Together, these studies suggest significant alterations in the gut microbiota of obese dogs compared to the healthy lean ones. However, due to the heterogeneity of these studies, including variations in factors such as age, sex, breed, nutrition level, and genetic factors, the conclusions drawn are inconsistent.

#### Gut microbiota alteration in obese cats

Similarly, the gut microbiota of obese cats is also dysbiotic. A metagenomic study of the gut microbiota of 8 obese cats and 8 healthy cats has found that the microbial diversity and the Firmicutes-to-Bacteroidetes ratio in obese cats are significantly reduced, while the abundance of *Bifidobacterium* sp., *Dialister* sp., *Olsenella provencensis* and *Campylobacter upsaliensis* are increased compared to the healthy control. Additionally, the quantity of *Fusobacteria, Erysipelotrichaceae* bacterium, and *Phascolarctobacterium succinatutens* is decreased in the obese cats’ microbiota [106]. Furthermore, a microbial study of 82 domestic cats has also shown an increase in *Bifidobacterium* abundance and a decrease in *Fusobacteria* abundance in obese cats, whereas the abundance of *Coriobacteriales* belonging to the Actinobacteria phylum was decreased compared to the lean ones [107]. Another study on the effects of dietary intervention on obese cats has shown that the abundance of Actinobacteria increases in obese cats during weight loss, primarily due to changes in *Bifidobacterium* sp. and *Collinsella* sp. Meanwhile, there is a decrease in bacterial infections in obese cats when they lose weight, mainly driven by the *Prevotella* spp. reduction. Within the Firmicute phylum, *Blautia*, *Dorea*, *Eubacterium*, *Oscillospira*, *Peptococcus*, and *Ruminococcus* increased whereas *Lactobacillus*, *Butyricicoccus*, and *Phascolarctobacterium* decreased overtime [108]. A 16S rRNA gene sequencing study on the feces of 24 cats (including eight obese cats) shows a significant decrease in the Firmicutes-to-Bacteroidetes ratio in obese cats. Interestingly, there are substantial increases in *Prevotella* while the proportions of *Blautia* and *Clostridium* decline [109]. A study involving 14 lean neutered cats and 17 obese neutered cats has found that the gut microbiota of obese cats is enriched with Firmicutes, Actinobacteria, Proteobacteria, and Planococcaceae incertae sedis. However, this study found no significant differences in the biodiversity index of microbiota between obese and lean cats [110]. Overall, in the gut microbiota of obese cats, there is an increase in the abundance of *Proteobacteria* and a decrease in the abundance of Actinobacteria. Additionally, unlike obese dogs, there is a decrease in the Firmicutes-to-Bacteroidetes ratio and a decline in the abundance of *Fusobacteria* in the gut microbiota of obese cats. The difference may be related to the variations in the intestinal structure and dietary habits of the two animals.

Taken together, gut microbiota plays vital roles in pet lipid metabolism. Bacteroidetes, Firmicutes, and Actinobacteria produce SCFAs by fermenting non-digestible carbohydrates (such as starch, inulin, cellulose, hemicellulose, pectin, and gum). These SCFAs provide energy to the intestinal epithelium and other tissues and support the intestinal defense mechanism by lowering the intestinal pH value. Furthermore, they influence the capacity for intestinal absorption and digestion, ultimately affecting obesity [111]. However, there are currently limited studies on the mechanism of intestinal microbiota regulating animal fat deposition. Metagenomics, metabolomics, and transcriptomics offer potential explanations for the interaction between these two factors. By utilizing these multi-omics research methods, we can further explore the mechanisms of intestinal microorganisms in pet lipid metabolism. Thus, we may identify potential signaling pathways and regulatory factors and discover new therapeutic and intervention strategies for controlling and regulating animal fat deposition.

#### The mechanisms of gut microbiota in regulating lipid metabolism and the potential role of the microbiota-gut-brain axis

The gut microbiota plays a crucial role in regulating the host’s lipid metabolism through various mechanisms such as the production of SCFAs, BA metabolism, hunger regulation, maintenance of intestinal barrier function, immune response modulation, and specific enzyme production. These processes impact energy expenditure and lipid absorption, potentially affecting the onset of metabolic diseases like obesity. Moreover, the dysregulation of the gut microbiota can impair the host's ability to process polysaccharides and absorb dietary lipids, leading to disruptions of liver and adipose tissue functions and initiating a cascade of metabolic disorders, including obesity, fatty liver, type 2 diabetes, and cardiovascular diseases [6]. However, the precise mechanisms underlying the interplay between gut microbiota and lipid metabolism require further investigation. Existing evidence has demonstrated that the gut microbiome can regulate lipid metabolism and subsequently impact body weight via the modulation of specific microbial metabolites, such as BAs, SCFAs, TMAO, tryptophan and indole derivatives [112–114]. For instance, TMAO can induce a high liver score of fatty liver hemorrhage syndrome by reducing the total BA content in the liver and increasing the total cholesterol [113]. These microbial metabolites engage in a cross-talk with the intestinal barrier and endocrine hormones, consequently regulating lipid metabolism through the microbiota-gut-brain axis.

**Fig. 4:**
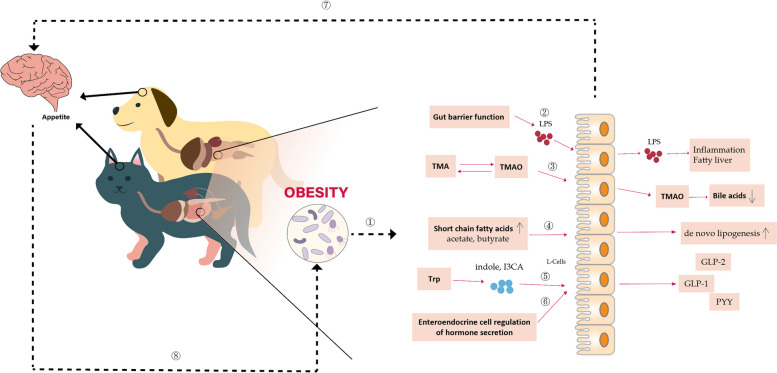
Why the gut microbiota plays a crucial role in the development of obesity. Gut flora in obese dogs and cats ① can affect lipid metabolism in several ways: ② LPS is increased in the intestines of obese dogs and cats and causes inflammation and fatty liver when it enters the bloodstream through a weakened intestinal barrier. ③ TMA produced by intestinal flora through the metabolism of methylamine-containing nutrients is converted to make TMAO, which promotes hepatic fat formation by affecting bile acids. ④ The gut flora of obese dogs and cats produces more SCFAS, such as acetate and butyrate, that promote de novo fat synthesis. ⑤ Tryptophan is metabolized by the intestinal microbiota to indole and its derivatives that can stimulate the production of GLP-1 by enteroendocrine L-cells and insulin secretion by pancreatic β-cells and serve as molecular signals to regulate food intake and appetite. ⑥ Gut flora alters GLP-1, GLP-2, and PYY secretion by regulating enteroendocrine cells (L cells). ⑦ Gut flora communicates with the brain through metabolic byproducts and neural pathways, influencing mood and behavior. ⑧ Brain modulates the composition and function of the gut flora through neuroendocrine responses and immune system alterations. LPS: lipopolysaccharide, GLP-1: glucagon-like peptide-1, GLP-2: glucagon-like peptide-2, PYY: peptide tyrosine tyrosine, Trp: tryptophan, I3CA: indole-3-carboxylic acid

Recently, it has also been suggested that the gut microbiota communicates directly with the brain through neurological pathways, such as the vagus nerve, and affects brain function via hormonal and immune pathways. Microbiota-derived SCFAs can regulate the host’s energy balance and mood through specific receptors, impacting the synthesis of key neurotransmitters like serotonin [115]. Moreover, the gut microbiota potentially modulates the expression of brain-derived neurotrophic factor (BDNF), which is crucial for neuronal growth and survival. In return, the brain influences the composition and function of the gut microbiota through neuroendocrine stress responses and regulates gastrointestinal motility and secretion via the autonomic nervous system [115–117]. The brain also modulates the gut microbiota by affecting the activity of immune cells and immune responses, while controlled behaviors and dietary habits directly impact these microbes [118]. These physiological processes described are all normal physiological processes for animals within the normal range. However, when they are imbalanced or excessive, they can all potentially induce metabolic and obesity issues in animals, which is why gut microbiota is so crucial for the development of obesity in animals (Fig. 4).

## The significance of diverse nutritional requirements in the management of obesity in dogs and cats

### Variations in nutrient requirements between dogs and cats

In the wild, cats consume prey that is rich in proteins and contains moderate amounts of fat and minimal quantities of carbohydrates [119]. Consequently, they have evolved as a carnivore species, possessing a metabolic adaptation that allows for a higher rate of protein metabolism and lower utilization of carbohydrates compared to dogs and other omnivores [120]. In contrast, in canines, approximately 40% to 70% of ingested glucose during a meal is absorbed by the liver via the portal circulation [121]. Whereas the feline hepatic glucose extraction is comparatively low due to the absence of the hepatic enzyme glucokinase [122]. The lack of this enzyme reflects, to a certain extent, the relatively limited contribution of dietary carbohydrates in maintaining blood glucose levels in feline physiology. The evolutionary divergence in energy metabolism determines that feline species refrain from down-regulating amino acid catabolic enzymes, even when dietary protein is limited. It implies that cats may have to engage in the breakdown of lean mass to generate amino acids for hepatic gluconeogenesis, thereby supporting the maintenance of circulating glucose levels [123]. The significant differences in protein requirements between cats and omnivorous species, such as dogs, further emphasize this important metabolic distinction. For instance, while the protein requirement for kittens is 1.5 times higher than that of young individuals from other species, adult cats require 2 to 3 times more protein in their diet than mature individuals from omnivorous taxa [124].

### Nutritional and health risks of restricted feeding

By implementing a restricted diet, it is possible to maintain a healthy weight for both dogs and cats, preventing obesity. However, it should be noted that limited food intake in cats can potentially lead to lipid deposition disorder [57]. Energy restriction may prompt the mobilization of FA. Still, insufficient choline intake resulting from the limitation can compromise FA transport from the liver and reduce FA oxidation, leading to fat accumulation and eventually hepatic lipid deposition [57]. In clinical practice, it is generally considered safe for cats to lose weight at a rate of 0.5%–2% of their initial body weight per week [25]. Rapid weight loss beyond this recommended range can increase the risk of feline hepatic lipidosis [125]. However, currently there is no standardized formula in the pet industry for calculating how to restrict energy intake [25, 126–128]. Therefore, the sector must unify and establish a scientifically-based program for energy restriction in the future.

### Nutritional interventions targeting gut microbiome

#### Dietary intervention

Variations in macronutrient ratios have been reported to significantly impact the gut microbiota composition of animals, including cats [129]. In dogs, the composition and function of the gut microbiota can be selectively altered by diets with varying macronutrient ratios, especially during obesity [130, 131]. Weight-loss foods are often characterized to have a high protein, low carbohydrate, and low-fat content. Numerous studies conducted on humans, mice, dogs, and cats have demonstrated the potential benefits of these diets, including increased satiety, decreased body fat, and maintenance of lean body weight [132–135]. Notably, the reason for the elevated protein inclusion in the weight loss formulations is not solely for the satiating effect but rather to ensure animals continue to receive adequate protein intake while the total energy consumption is reduced, thus maintaining a high protein-to-energy ratio (PER) [136, 137]. Since pets depend on their owners for food, maintaining a high PER is critical, especially during weight management programs. This approach allows pets to consume significantly less energy while obtaining sufficient protein. In addition, the provision of protein can also facilitate fat reduction and aid in preventing weight rebound [130]. Despite the potential benefits, excess protein intake has been associated with adverse effects on the gut microbiota, altering the metabolic byproducts such as causing SCFAs reduction or inducing TMAO production [138–140] and promoting intestinal inflammation [141]. A study of dogs found that in contrast to increased *Bacteroides uniformis* and *Clostridium butyricum* promoted by a low-protein, high-carbohydrate diet, the growth of a high-protein, low-carbohydrate diet increased the abundance of *Clostridium hiranonis*, *Clostridium perfringens*, and *Ruminococcus gnavus*, all of which are potential pathogens. Additionally, the high-protein, low-carbohydrate diet resulted in a decrease in the Bacteroidetes-to-Firmicutes ratio and an increase in the *Bacteroides*-to-*Prevotella* ratio [130].

High-carbohydrate diets may contribute to obesity. Excessive consumption of carbohydrates and exceptionally high simple sugar intake can rapidly increase blood glucose levels, stimulating insulin secretion and promoting fat production. Furthermore, a high-sugar diet can induce insulin resistance, resulting in hyperinsulinemia and high blood pressure [142, 143]. However, current research cannot establish a causal relationship between a high-carbohydrate diet and pet obesity. A study involving neutered cats reveals that high-fat diets resulted in more significant weight gain than a high-carbohydrate diet [144]. This implies that providing low-energy density feed may offer a viable strategy for preventing obesity.

Indeed, fat is one of the three macronutrients, characterized by its high energy density and compact size. A high-fat diet can modify the composition of the gut microbiota and impair the intestinal mucosa, contributing to the development of obesity [145]. A study of healthy adult dogs shows that high-fat, low-carbohydrate diets with different percentages of canola oil inclusion resulted in linear increases of Tenericutes, Spirochaetes, Fibrobacteres, and Planctomycetes according to fat levels. In contrast, *Prevotellaceae*, *Catenibacterium*, *Alloprevotella*, and bacteria belong to the Lachnospiraceae family, and the genus *Bacteroides* showed a linear decrease in abundance with increasing fat content in the diet. It is speculated that bacterial populations that increase dietary fat levels may utilize fat or bile as a key metabolic substrate and vice versa [146]. In addition, studies have shown that specific gut microbes, such as *Bifidobacterium*, can directly or indirectly regulate the absorption, bioavailability, and biotransformation of omega-3 polyunsaturated fatty acids (PUFAs), further affecting FA intake and their functions. PUFA-derived metabolites produced by the gut microbiota may be a new type of active metabolite, capable of exerting anti-obesity and anti-inflammatory effects through various mechanisms [147]. Another study has also shown that the gut microbiota can convert dietary PUFAs, such as 10-hydroxy-cis-12-octadecenoic acid (HYA) derived from linoleic acid, into a variety of metabolites. Furthermore, supplementation with HYA can alleviate obesity induced by a high-fat diet in mice. This effect is independent of arachidonic acid (AA)-mediated adipose inflammation and improves metabolic conditions through free fatty acid receptors. By producing metabolites such as HYA, the gut microbiota helps to regulate the host's energy balance and insulin sensitivity, thereby enhancing resistance to metabolic disorders [148].

#### Probiotics

In addition to macronutrient inclusion, supplementation of probiotics, prebiotics, synbiotics and/or postbiotics may also be approaches of interest in managing obesity in pets (Table 1). Probiotics are defined as "living microorganisms that, when ingested in adequate amounts, confer health benefits to the host" [149]. Recent studies have demonstrated the significant role of probiotics in improving gut health. In a study of mice fed a high-fat diet, *Lactobacillus plantarum* K50 administration reduced fat content in adipose tissue and liver and improved gut microbial composition [150]. Furthermore, studies have demonstrated the ability of probiotic supplementation, particularly with *Lactobacillus* and *Bifidobacterium*, to effectively lower weight gain induced by a high-fat diet in obese mice and their blood glucose and lipid levels [151]. Moreover, *Lactobacillus fermentum* CECT5716 has ameliorated high-fat diet-induced obesity in mice by restoring the gut microbiota to its original normal state [152]. Studies suggest that probiotics exert a regulatory effect on gut microbiota, potentially through immune interactions, achieving weight loss goals in obese patients. Therefore, using probiotics may also improve the body fitness of pets [149].

**Table 1:** Studies on probiotics/prebiotics/synbiotics/postbiotics affect body weight in obese pets

Object of study	Intervention	ME	Duration of study	Key findings	Reference
Cats	Probiotics	The control group: 3,873 kcal/kg; The probiotic group: 3,873 kcal/kg + 4 kcal/g	18 weeks	No significant effect on food intake, body weight, body composition or metabolic parameters	[153]
Dogs	Probiotics	The control group: 357.0 kcal/100 g; The HFD group: 483.0 kcal/100 g	30 d	Decreased body weight, improved obesity phenotype, and lowered blood levels of TG, CHO, and LDL-C	[154]
Dogs	Probiotics		1 week	Increased lactic acid bacteria decreased total lipids and the total protein	[155]
Dogs	Prebiotics	The control group:16.19 kJ/g	14 d	Regulates gut microbiota, metabolites, and BA pool	[156]
Dogs	Synbiotics	-	10 weeks	Decreased body weight, increased microbial diversity, and abundance of carbohydrate and lipid metabolism	[157]
Dogs	Postbiotics	Control: 3,569 kcal/kg; 1% Fiber Bundle: 3,631 kcal/kg; 2% Fiber Bundle: 3,567 kcal/kg; 4% Fiber Bundle: 3,588 kcal/kg	59 d	Bacteria in the large intestine switched from digesting mainly protein to digesting mainly carbohydrates	[158]
Cats	Postbiotics	Control: 4,066 kcal/kg; 1% Fiber Bundle: 4,049 kcal/kg; 2% Fiber Bundle: 4,031 kcal/kg; 4% Fiber Bundle: 4,052 kcal/kg	59 d	Increased several compounds associated with beneficial health effects and decreased some compounds that indicate the breakdown of protein	[159]

Indeed, a study of high-fat-induced obesity in canines has demonstrated that *Enterococcus faecium* IDCC 2102 and *Bifidobacterium lactis* IDCC 4301 effectively promoted the proliferation of beneficial commensal bacteria in the intestinal tract. These probiotics activated glycolysis and pyruvate metabolism, enhancing energy metabolism and curbing weight gain in obese animals [154]. Marcináková et al. [155] also reported that oral administration of probiotic *Enterococcus faecium* strain EE38 decreased the total lipids and protein levels in blood from 8 out of 11 healthy dogs, where cholesterol levels reached physiological levels. *Enterococcus faecium* SF68 is an EU-approved pet probiotic commonly used in research on pet diseases [160, 161]. However, a study conducted by Kathrani et al. [153] on naturally occurring obesity in cats, free from specific pathogens, shows that this probiotic did not influence animal body weight, composition, or metabolic parameters. Notably, this study's intervention duration was short. Probiotic products for dogs and cats are trendy nowadays, where optimal effects are often found with host-derived probiotic preparations [162]. Indeed, it is suggested that probiotics of host origin may exhibit higher colonization efficacy in the intestines of the corresponding species [163]. Thus, using beneficial microorganisms isolated from dogs and cats may better address the gastrointestinal needs of obese animals.

#### Prebiotics/synbiotic

Prebiotics are substances that can be selectively utilized by intestinal microorganisms and benefit the host’s health. They encompass functional oligosaccharides, non-starch polysaccharides, resistant starch, proteins, plant extracts etc. [164]. A study involving nine overweight beagles reveals that the commercial prebiotic inulin-type fructans increased the SCFA production and the relative abundance of Eubacterium (affiliated with Firmicutes), and decreasing the relative abundance of some Proteobacteria, ultimately improving the glucose and insulin incremental area under the curve of the overweight animals [156]. There have been reports on the positive effects of short-chain fructo-oligosaccharides on reducing fat deposits and regulating specific bacteria in animals [165]. A study on high-fat feeding in mice found that galactooligosaccharides addition restored the intestinal microbial dysbiosis by increasing the reduction of beneficial microbes such as *Akkermansia muciniphila*, *Bacteroides acidifies*, *Lactobacillus gasseri* and *Bifidobacterium pseudopodium*, which was closely related to the improved body weight gain, insulin resistance, and lipid metabolism [166]. Research has also found that bamboo shoot fiber effectively prevents obesity in mice by modulating the intestinal microbial community [167]. Ingesting *Ganoderma lucidum* water extract can reduce weight and inflammation and alleviate insulin resistance in mice on a high-fat diet [156]. These studies imply that prebiotics may also be used in managing pet obesity. However, most prebiotic studies were conducted with healthy cats and dogs [14]. One study of 12 healthy adult domestic shorthair cats found that resistant starch promoted the intestinal growth of Actinobacteria and butyrate production while reducing the blood cholesterol levels in cats [168]. Similarly in canines, prebiotic fibers are also reported to enrich fiber-fermenting microbes and increase SCFA production [14, 169].

Synbiotics are mixtures comprising live microorganisms and substrate(s) selectively utilized by intestinal microorganisms that confer health benefits on the host [170]. It is summarized that certain combinations of probiotics and prebiotics exhibit superior beneficial effects compared to products containing either probiotics or prebiotics alone in managing obesity and the associated gut microbiota dysbiosis [171–174]. In obese dogs, it is found that a synbiotic containing *Lactobacillus gasseri* BNR17 and a non-digestible maltodextrin, galacto-oligosaccharides, fructo-oligosaccharides, and polydextrose mixture can decrease body weight and subcutaneous fat levels at the third lumbar vertebra, enhance microbial diversity, and improve carbohydrate and lipid metabolism [157]. There is a lack of primary experimental data on probiotics and prebiotics compatibility, which warrants further investigations.

#### Postbiotics

Postbiotics are defined as non-living microorganisms and their components that could confer health benefits to the host [175]. Effective postbiotics must contain inactive microbial cells or cell components, with or without metabolites, contributing to observed health benefits. A study of 48 healthy dogs reveals that feeding fiber and polyphenolic components resulted in a shift of gut microbiota in the colon from primarily digesting proteins to primarily digesting carbohydrates, thus producing potential postbiotic compounds [158]. Similarly, another study on postbiotics in cats also indirectly confirmed the potential of certain positive postbiotics to alleviate obesity in pets [176]. Conversely, dietary fats significantly affect the gut microbiota metabolism and potential postbiotic production. Specific FA, such as medium-chain triglycerides and long-chain polyunsaturated FA, can regulate the metabolic activities of the microbial community, leading to changes in the levels of various lipid classes. Furthermore, the intake of specific long-chain polyunsaturated FA can alter the concentrations of gut microbiome-derived postbiotics, such as indoles/indolic sulfates and phenols/phenolic sulfates, thereby further influencing health, obesity and related metabolic diseases [176, 177].

#### Fecal microbiota transplantation (FMT)

Fecal microbiota transplantation has emerged as a technique in medicine in recent years and has undergone clinical applications in treating gut microbiota-related diseases in humans and dogs [178, 179]. This therapy involves transferring a donor's healthy microbial community using a fecal preparation into the recipient's gut, thereby reconstructing their gut microbiota composition and achieving therapeutic goals. Symbiotic bacteria are considered the primary practical components of FMT. However, other elements such as viruses, bacterial fragments, proteins, antimicrobial compounds, metabolites, oligonucleotides, and shed cells from the donor also play essential roles [180]. Previous research has investigated the indications of FMT in dogs, showing positive regulatory effects on disease treatment. For instance, dogs treated with FMT recovered quicker from acute diarrhea than those treated with metronidazole. The FMT-treated dogs exhibited a lower abundance of *E. coli* and *Streptococcus* spp. in their gut microbiota and an overall more diverse community [178]. FMT has also been demonstrated to be effective in treating diseases such as *Clostridium difficile* infection (CDI) [181], inflammatory bowel disease (IBD) [182–184], canine parvovirus (CPV) enteritis [185], and acute hemorrhagic diarrhea syndrome (AHDS) [186, 187]. However, research using FMT in cats is relatively limited compared to dogs. Furmanski et al. [188] reported a case study of a cat with ulcerative gastroenteritis unresponsive to conventional treatments. After undergoing two FMT procedures, the cat’s condition was gradually stabilized, and its bowel movements were normalized [188]. Another case study found FMT improved vomiting and diarrhea in a 6-year-old cat [189]. Furthermore, a study evaluating the changes in fecal microbiota after FMT in cats confirmed the efficacy of orally administered FMT capsules in improving chronic colitis [190].

Unfortunately, there is a lack of research exploring the effects of FMT on obesity in pets. Nevertheless, considering the findings from human FMT studies, it is reasonable to speculate that FMT may play a positive role in managing pet obesity. A study in mice reveals that FMT had mitigated obesity induced by a high-fat diet [191]. The effects may be attributed to the significant alteration of the composition and structure of the gut microbiota following FMT, including an increased abundance of *Romboutsia* and *Lactobacillus*. Interestingly, changes in the composition of the gut microbiota would not only affect the process of intestinal lipid metabolism but also impact the levels of m^6^A, a methylation modification found on RNA molecules that plays a crucial role in regulating gene expression. Variations in the gut microbiota, by modulating m^6^A levels, may alter the absorption and excretion of fats, thereby reducing the intestinal uptake of lipids, enhancing lipid excretion, and ultimately boosting the host’s resistance to obesity [192, 193]. In a study by Ridaura et al. [194], they cohabitated mice carrying the gut microbiota from obese twins with mice carrying the gut microbiota from lean twins. The researchers observed that mice with ‘obese microbiota’ exhibited inhibition of fat growth, body weight, and the development of obesity-related metabolic phenotypes. Additionally, the microbiota metabolic profile of the obese mice was eventually transformed into a state similar to that of lean mice. These promising studies suggest that FMT may have become a valuable approach for testing pet obesity.

Although FMT therapy has emerged as a potential alternative to conventional drug treatments in veterinary clinics, it has also encountered several challenges. One of the critical issues is the stringent screening process required for donor feces to prevent infectious disease transmission. Moreover, a notable obstacle is the absence of a dedicated “fecal bank” for pets, which adds complexity and expense to the screening process. While trying to establish such banks, they would face some difficulties. Firstly, the infrequent use of FMT in the current veterinary clinics could result in the long-term storage of fecal material, potentially compromising its efficacy. Additionally, research on pet FMT is limited, failing to account for the potential impacts of breed, size, age, and physiological status on its effectiveness. Further research on pet FMT is essential to understand its mechanisms and applications [178] comprehensively.

In general, significant gaps remain in understanding the mechanisms of gut microbiota in controlling obesity and identifying potential targets for obesity prevention. Various factors influence obesity and the complex interactions among gut microbiomes and host metabolism. Interventions like fecal microbiota transplantation, probiotics, and prebiotics could quickly address gut microbiota imbalance. However, a drawback is that once the intervention ceases and the patient resumes a normal diet, the gradual disappearance of the acquired beneficial microbiota may occur. Therefore, the combination of multiple strategies is vital for sustaining a stable gut microbiota and could have a synergistic effect in regulating the body’s metabolism. This entails reducing the consumption of high-energy-density foods, modifying the macronutrient composition (e.g., high-protein, low-fat, and high-fiber diets), and incorporating prebiotics and probiotics [153, 195–197]. Nevertheless, current research indicates that approximately half of obese cats and dogs regain weight following a weight loss plan. This emphasizes the need for personalized nutrition plans to effectively control weight and achieve precise nutritional effects.

## Conclusion

Over the past decades, a high prevalence of obesity has been found in dogs and cats. It is clear that obesity is associated with several related chronic diseases in these animals. While previous studies have shed light on the role of lipid metabolism in obesity, the process of lipid metabolism in companion animals is still poorly understood. This study clarifies the importance of lipid metabolism and its relationship with gut microbiota. Additionally, this review discusses nutritional interventions for managing obesity in dogs and cats, specifically focusing on the gut microbiome. These interventions involve dietary modifications, probiotics, prebiotics/synbiotics, postbiotics, and FMT. These interventions show potential for enhancing lipid metabolism in obese dogs and cats and offer valuable scientific evidence and practical nutritional strategies for clinical practice.

## Data Availability

Not applicable.

## Abbreviations

AHDS: Acute hemorrhagic diarrhea syndrome
BA: Bile acids
BCS: Body condition score
BDNF: Brain-derived neurotrophic factor
CETP: Cholesterol ester transfer protein
CHO: Cholesterol
DEXA: Dual-energy X-ray absorptiometry
DM: Diabetes mellitus
DNL: De novo lipogenesis
FA: Fatty acid
FFA: Free fatty acid
FMT: Fecal microbiota transplantation
HDL: High-density lipoproteins
HMW: High molecular weight
HYA: 10-Hydroxy-cis-12-octadecenoic acid
LDL: Low-density lipoproteins
NEFA: Non-esterified fatty acid
PUFA: Polyunsaturated fatty acid
SCFA: Short-chain fatty acid
TG: Triglyceride
TMAO: Trimethylamine oxide
VLDL: Very low-density lipoproteins
